# Supplementary material for: Exploratory study reveals far reaching systemic and cellular effects of verapamil treatment in subjects with type 1 diabetes
Source: Nat Commun. 2022 Mar 3;13:1159. doi: 10.1038/s41467-022-28826-3 (PMC8894430; doi:10.1038/s41467-022-28826-3)
Supplement: Supplementary file 1 — Supplementary Information [file 41467_2022_28826_MOESM1_ESM.pdf]

Supplementary Table 1: Baseline Characteristics of Study Subjects

	**Verapamil T1D**[1]<br>n=9<br>[n=5 for LC-MS/MS & qPCR] [3]	**Control T1D**[1]<br>n=6<br>[n=5 for LC-MS/MS & qPCR] [3]	(Healthy)[2]<br>n=9<br>[n=7 for qPCR] [3]
**Age** (years)	30.4 ± 2.4<br>[25.6 ± 2.7]	27.8 ± 2.8<br>[28.4 ± 3.3]	*(31.0 ± 2.0)*<br>[30.6 ± 2.6]
**Gender**	5M/4F<br>[3M/2F]	3M/3F<br>[2M/3F]	*(3M/6F)*<br>[3M/4F]
**Race**	8W/1AA<br>[5W]	6W<br>[5W]	*(6W/1AA/2A)*<br>[4W/1AA/2A]
**BMI** (kg/m^2)	24.1 ± 1.1<br>[24.3 ± 1.5]	24.8 ± 1.3<br>[24.5 ± 1.5]	*(26.4 ± 0.8)*<br>[25.8 ± 0.9]
**HbA1C** (%)	6.5 ± 0.3<br>[6.8 ± 0.5]	6.9 ± 0.4<br>[7.1 ± 0.4]	*(5.2 ± 0.1)*<br>[5.2 ± 0.1]

[1]*All study subjects had been diagnosed with T1D within 3 months and were positive for glutamic acid decarboxylase (GAD65) autoantibodies, except for one who was positive for microinsulin (MIAA) and islet cell (ICA) autoantibodies.*
[2]*Characteristics of healthy, non-diabetic subjects whose blood samples were used for comparison are shown in ( )*
[3]*Subset of study subjects whose serum was used for LC-MS/MS and whose PBMCs were used for qPCR are shown in [ ]*
W: White; AA: African American; A: Asian; BMI: Body Mass Index

Supplementary Table 2: Relative Abundance Levels in Serum (Log2transformed)

Protein	*Gene*	P-value	Baseline		Year 1	
			Placebo	Verapamil	Placebo	Verapamil
**sp|P10645|CMGA_HUMAN**	*CHGA*	0.00124	0.06848	0.0931	0.18352	-0.3396
sp|Q92954|PRG4_HUMAN	*PRG4*	0.00125	0.04164	-0.0539	-0.03562	0.18814
sp|P80188|NGAL_HUMAN	*LCN2*	0.00134	-0.02849	-0.08538	-0.1248	0.16444
sp|Q7Z5L0|VMO1_HUMAN	*VMO1*	0.00234	-0.42004	0.39604	0.01236	0.1014
sp|O14791|APOL1_HUMAN	*APOL1*	0.00293	0.04936	0.02484	-0.1626	0.2364
sp|Q6FHJ7|SFRP4_HUMAN	*SFRP4*	0.00439	0.0136	-0.00796	-0.12054	0.416
sp|P14780|MMP9_HUMAN	*MMP9*	0.00492	0.03205	-0.136524	-0.07446	0.2043
sp|P06727|APOA4_HUMAN	*APOA4*	0.00554	-0.17769	0.0612	0.26278	-0.206203
sp|P11021|BIP_HUMAN	*HSPA5*	0.00637	-0.03898	0.002726	0.01426	-0.033842
sp|P02788|TRFL_HUMAN	*LTF*	0.00708	0.06752	-0.19332	-0.14269	0.18546
sp|Q8NI99|ANGL6_HUMAN	*ANGPTL6*	0.00736	0.3234	-0.08554	0.11018	0.06312
sp|Q9UKU6|TRHDE_HUMAN	*TRHDE*	0.00851	-0.1899	0.081	-0.34914	0.3308
sp|P05164|PERM_HUMAN	*MPO*	0.00953	0.0352	-0.07654	-0.13396	0.26272
sp|P40197|GPV_HUMAN	*GP5*	0.0102	0.16982	-0.01604	0.082306	0.108852
sp|P04004|VTNC_HUMAN	*VTN*	0.0104	0.00702	0.204238	-0.11748	0.25182
sp|P60033|CD81_HUMAN	*CD81*	0.0119	0.06958	-0.1002	0.07636	-0.7528
sp|Q96PD5|PGRP2_HUMAN	*PGLYRP2*	0.0139	0.2038	0.1052	0.13522	0.21682
sp|P07858|CATB_HUMAN	*CTSB*	0.0148	-0.04898	0.0128	0.16254	-0.0276
sp|Q8WVN6|SCTM1_HUMAN	*SECTM1*	0.016	0.11646	-0.298678	0.3066	-0.6522
sp|P0DMV8|HS71A_HUMAN	*HSPA1A*	0.0167	0.04052	-0.05426	-0.32212	0.13
sp|O14786|NRP1_HUMAN	*NRP1*	0.0171	-0.05514	0.0436	-0.00526	-0.0884
sp|P02652|APOA2_HUMAN	*APOA2*	0.0175	0.12328	-0.08732	-0.05942	0.22806
sp|O95998|I18BP_HUMAN	*IL18BP*	0.0178	0.0276	-0.196768	0.06438	-0.39682
sp|P24592|IBP6_HUMAN	*IGFBP6*	0.0182	-0.00122	-0.20182	0.094796	-0.403
sp|P61158|ARP3_HUMAN	*ACTR3*	0.02	-0.23154	0.17667	-0.11482	-0.26824
sp|P28827|PTPRM_HUMAN	*PTPRM*	0.0229	0.04594	0.098	0.10762	-0.01537
sp|P18065|IBP2_HUMAN	*IGFBP2*	0.023	-0.0666	-0.3107	0.2856	-0.3918
sp|O95980|RECK_HUMAN	*RECK*	0.0237	-0.04358	0.16091	0.008072	-0.07366
sp|O00462|MANBA_HUMAN	*MANBA*	0.0241	0.18928	0.018764	0.023378	0.041358
sp|Q15113|PCOC1_HUMAN	*PCOLCE*	0.0258	0.19766	-0.09564	0.3402	-0.201516
sp|P05156|CFAI_HUMAN	*CFI*	0.0263	0.08389	0.01388	-0.00104	0.0605
sp|Q9Y6Z7|COL10_HUMAN	*COLEC10*	0.0268	0.05626	0.12426	0.13228	0.050476
sp|P01714|LV319_HUMAN	*IGLV3-19*	0.0294	0.2772	0.08014	0.4152	-0.19688
sp|P26447|S10A4_HUMAN	*S100A4*	0.0299	0.0234	0.07784	0.19582	-0.1978
sp|P13501|CCL5_HUMAN	*CCL5*	0.0304	0.1606	-0.2818	-0.23198	-0.16252
sp|Q8IYS5|OSCAR_HUMAN	*OSCAR*	0.0304	0.3306	-0.06058	0.38148	-0.257396
sp|P10412|H14_HUMAN	*H1-4*	0.031	-0.00185	-0.2648	-0.5276	-0.14708
sp|Q8N6C8|LIRA3_HUMAN	*LILRA3*	0.0325	0.10776	-0.0668	0.16114	-0.2232
sp|Q10471|GALT2_HUMAN	*GALNT2*	0.035	0.02464	0.152514	-0.12936	0.370482
sp|O75144|ICOSL_HUMAN	*ICOSLG*	0.0354	0.17966	0.14062	0.07296	0.1908
sp|Q02487|DSC2_HUMAN	*DSC2*	0.0361	-0.06798	0.0448	-0.02243	-0.08911
sp|P01024|CO3_HUMAN	*C3*	0.0364	0.11928	0.03756	-0.07992	0.2122
sp|P14314|GLU2B_HUMAN	*PRKCSH*	0.0381	-0.17948	0.18932	-0.11918	0.03404
sp|Q58FF3|ENPLL_HUMAN	*HSP90B2P*	0.0403	-0.1878	0.08606	-0.1415	-0.1124
sp|P13796|PLSL_HUMAN	*LCP1*	0.0408	0.14034	0.03244	0.07042	0.04378
sp|Q16769|QPCT_HUMAN	*QPCT*	0.0428	0.05212	-0.02816	-0.04822	0.21076
sp|P35542|SAA4_HUMAN	*SAA4*	0.0436	0.16974	-0.02206	-0.13044	0.0921
sp|P27169|PON1_HUMAN	*PON1*	0.0441	0.00255	0.003	-0.16072	0.107836
sp|Q01459|DIAC_HUMAN	*CTBS*	0.046	0.13946	-0.10516	0.0951	-0.02914
sp|Q08380|LG3BP_HUMAN	*LGALS3BP*	0.0469	0.05166	-0.0788	-0.11942	0.1034
sp|P61626|LYSC_HUMAN	*LYZ*	0.047	0.12044	0.11882	0.04794	0.124
sp|P09619|PGFRB_HUMAN	*PDGFRB*	0.0478	0.01076	0.10214	0.05788	0.0264
sp|P55268|LAMB2_HUMAN	*LAMB2*	0.0492	0.0862	-0.00802	-0.19332	0.0093

(Bold: top serum protein affected by verapamil; P-value: Two-tailed nested model)

Supplementary Table 3:
GO Biological Processes – Human Serum Proteomics

**Term** (enriched in response to verapamil)	**Overlap**	**P-value**	**Adjusted P-value**	**Odds Ratio**	**Combined Score**
neutrophil degranulation (GO:0043312)	12/481	3.56E-09	1.24E-06	12.2	236.5
neutrophil activation involved in immune response (GO:0002283)	12/485	3.91E-09	1.24E-06	12.1	233.3
neutrophil mediated immunity (GO:0002446)	12/488	4.19E-09	1.24E-06	12.0	231.0
positive regulation of smooth muscle cell migration (GO:0014911)	4/17	1.02E-07	2.27E-05	125.2	2015.2
regulation of smooth muscle cell migration (GO:0014910)	4/20	2.06E-07	3.68E-05	101.7	1565.4
innate immune response (GO:0045087)	8/302	1.21E-06	1.71E-04	11.9	161.9
cellular protein metabolic process (GO:0044267)	9/417	1.34E-06	1.71E-04	9.8	132.4
negative regulation of cellular component organization	5/80	2.23E-06	2.48E-04	27.6	359.2
defense response to bacterium (GO:0042742)	6/176	6.95E-06	6.88E-04	14.9	176.4
positive regulation of cellular metabolic process (GO:0031325)	5/105	8.51E-06	7.49E-04	20.7	241.4
regulation of complement activation (GO:0030449)	4/50	9.25E-06	7.49E-04	35.3	409.4
regulation of immune effector process (GO:0002697)	4/53	1.17E-05	8.63E-04	33.1	376.5
regulation of humoral immune response (GO:0002920)	4/54	1.26E-05	8.63E-04	32.5	366.5
positive regulation of cell migration (GO:0030335)	6/269	7.57E-05	4.81E-03	9.6	90.7

(Overlap: # of enriched genes in dataset/ # of genes in term; P-value: Fischer exact test; Adjusted p-value: Benjamini-Hochberg)

Supplementary Table 4:
Characteristics of Study Subjects at Start of Year 2

	**Verapamil T1D** n=5	**Disc V T1D** n=4	**Control T1D** n=6
**Age** (years)	31.6 ± 3.3	31.3 ± 3.6	28.8 ± 2.8
**Gender**	4M/1F	1M/3F	3M/3F
**Race**	4W/1AA	4W	6W
**BMI** (kg/m^2)	25.9 ± 1.5	23.4 ± 2.0	25.9 ± 1.4
**HbA1C** (%)	6.3 ± 0.2	6.2 ± 0.2	7.2 ± 0.4

Supplementary Table 5:
GO Biological Processes – Human Islet Transcriptomics

**Term** (enriched in response to verapamil)	**Overlap**	**P-value**	**Adjusted P-value**	**Odds Ratio**	**Combined Score**
extracellular matrix organization (GO:0030198)	53/300	3.13E-25	9.57E-22	7.3	410.0
extracellular structure organization (GO:0043062)	38/216	2.46E-18	2.96E-15	7.1	286.6
external encapsulating structure organization (GO:0045229)	38/217	2.90E-18	2.96E-15	7.0	283.8
proteolysis (GO:0006508)	36/287	9.38E-13	7.18E-10	4.7	130.6
neutrophil mediated immunity (GO:0002446)	44/488	2.27E-10	1.39E-07	3.3	72.6
neutrophil degranulation (GO:0043312)	43/481	4.78E-10	2.44E-07	3.2	69.4
neutrophil activation involved in immune response (GO:0002283)	43/485	6.17E-10	2.70E-07	3.2	68.0
positive regulation of cell population proliferation (GO:0008284)	42/474	1.00E-09	3.83E-07	3.2	66.3
platelet degranulation (GO:0002576)	20/125	1.61E-09	5.04E-07	6.1	124.3
collagen fibril organization (GO:0030199)	17/89	1.64E-09	5.04E-07	7.6	153.4
regulated exocytosis (GO:0045055)	23/180	8.76E-09	2.44E-06	4.7	87.8
regulation of fibroblast proliferation (GO:0048145)	12/46	1.01E-08	2.59E-06	11.3	207.4
positive regulation of cell migration (GO:0030335)	28/269	2.20E-08	5.19E-06	3.8	66.5
aerobic electron transport chain (GO:0019646)	14/70	2.45E-08	5.36E-06	8.0	140.2
mitochondrial ATP synthesis coupled electron transport (GO:0042775)	14/71	2.96E-08	6.05E-06	7.9	136.2
hexose biosynthetic process (GO:0019319)	11/44	6.81E-08	1.30E-05	10.6	175.3
positive regulation of fibroblast proliferation (GO:0048146)	9/28	9.89E-08	1.71E-05	15.1	242.9
positive regulation of cell motility (GO:2000147)	24/221	1.01E-07	1.71E-05	3.9	63.4
vasoconstriction (GO:0042310)	6/11	3.44E-07	5.08E-05	38.0	565.5
gluconeogenesis (GO:0006094)	10/41	3.48E-07	5.08E-05	10.3	152.7
glucose metabolic process (GO:0006006)	12/62	3.58E-07	5.08E-05	7.7	113.6
positive regulation of endothelial cell migration (GO:0010595)	14/86	3.65E-07	5.08E-05	6.2	92.2
negative regulation of endopeptidase activity (GO:0010951)	12/63	4.30E-07	5.49E-05	7.5	110.0
negative regulation of peptidase activity (GO:0010466)	12/63	4.30E-07	5.49E-05	7.5	110.0
vascular associated smooth muscle contraction (GO:0014829)	5/8	1.44E-06	1.76E-04	52.7	708.8
regulation of cell migration (GO:0030334)	32/408	1.54E-06	1.81E-04	2.8	36.9
regulation of endopeptidase activity (GO:0052548)	12/71	1.65E-06	1.87E-04	6.5	86.4
cobalamin metabolic process (GO:0009235)	7/21	2.07E-06	2.27E-04	15.8	207.4
negative regulation of blood coagulation (GO:0030195)	9/40	2.81E-06	2.92E-04	9.2	117.9
positive regulation of angiogenesis (GO:0045766)	15/116	2.91E-06	2.92E-04	4.7	60.5
tetrapyrrole metabolic process (GO:0033013)	7/22	2.96E-06	2.92E-04	14.8	188.3
prostaglandin metabolic process (GO:0006693)	8/31	3.34E-06	3.20E-04	11.0	139.2
basement membrane organization (GO:0071711)	6/16	5.22E-06	4.73E-04	19.0	231.0
regulation of angiogenesis (GO:0045765)	20/203	5.25E-06	4.73E-04	3.5	42.7
canonical glycolysis (GO:0061621)	7/24	5.69E-06	4.84E-04	13.1	157.6
glucose catabolic process to pyruvate (GO:0061718)	7/24	5.69E-06	4.84E-04	13.1	157.6
positive regulation of epithelial cell proliferation (GO:0050679)	15/123	6.07E-06	5.02E-04	4.4	53.3
homotypic cell-cell adhesion (GO:0034109)	9/44	6.53E-06	5.26E-04	8.2	97.5
glycolytic process through glucose-6-phosphate (GO:0061620)	7/25	7.69E-06	6.02E-04	12.3	145.1
positive regulation of smooth muscle cell migration (GO:0014911)	6/17	7.86E-06	6.02E-04	17.3	202.9
negative regulation of epithelial cell proliferation (GO:0050680)	11/72	1.23E-05	8.93E-04	5.7	64.9
positive regulation of secretion by cell (GO:1903532)	11/72	1.23E-05	8.93E-04	5.7	64.9
positive regulation of MAPK cascade (GO:0043410)	23/274	1.54E-05	1.10E-03	2.9	32.6
regulation of MAPK cascade (GO:0043408)	17/166	1.59E-05	1.11E-03	3.7	40.3
water-soluble vitamin metabolic process (GO:0006767)	11/76	2.08E-05	1.39E-03	5.4	58.1
mitochondrial electron transport, NADH to ubiquinone (GO:0006120)	8/39	2.09E-05	1.39E-03	8.2	88.2
glycolytic process (GO:0006096)	7/29	2.24E-05	1.45E-03	10.1	107.9
regulation of smooth muscle cell migration (GO:0014910)	6/20	2.27E-05	1.45E-03	13.6	145.0
regulation of endothelial cell proliferation (GO:0001936)	12/92	2.59E-05	1.62E-03	4.8	50.5
positive regulation of cellular process (GO:0048522)	39/625	2.79E-05	1.70E-03	2.2	22.7
regulation of cell population proliferation (GO:0042127)	45/764	2.83E-05	1.70E-03	2.0	21.3
antigen processing and presentation of endogenous peptide antigen via MHC class I via ER pathway (GO:0002484)	4/7	2.93E-05	1.70E-03	42.1	439.2
antigen processing and presentation of endogenous peptide antigen via MHC class I via ER pathway, TAP-independent (GO:0002486)	4/7	2.93E-05	1.70E-03	42.1	439.2
carbohydrate catabolic process (GO:0016052)	8/41	3.08E-05	1.75E-03	7.7	79.9
positive regulation of epithelial cell migration (GO:0010634)	12/94	3.22E-05	1.76E-03	4.7	48.2
cellular component disassembly (GO:0022411)	10/66	3.27E-05	1.76E-03	5.7	58.6
extracellular matrix disassembly (GO:0022617)	10/66	3.27E-05	1.76E-03	5.7	58.6
supramolecular fiber organization (GO:0097435)	26/351	3.89E-05	2.06E-03	2.6	26.1
pyruvate metabolic process (GO:0006090)	9/55	4.33E-05	2.22E-03	6.2	62.4
branching involved in blood vessel morphogenesis (GO:0001569)	5/14	4.40E-05	2.22E-03	17.6	176.1
regulation of MAP kinase activity (GO:0043405)	12/97	4.42E-05	2.22E-03	4.5	45.1
cellular protein metabolic process (GO:0044267)	29/417	4.51E-05	2.23E-03	2.4	24.1
sulfur compound biosynthetic process (GO:0044272)	13/113	4.67E-05	2.27E-03	4.1	41.3
positive regulation of MAP kinase activity (GO:0043406)	10/69	4.86E-05	2.32E-03	5.4	53.5
glycosaminoglycan catabolic process (GO:0006027)	9/56	5.02E-05	2.36E-03	6.1	60.2
regulation of endothelial cell apoptotic process (GO:2000351)	8/44	5.27E-05	2.44E-03	7.0	69.4
regulation of ERK1 and ERK2 cascade (GO:0070372)	20/238	5.37E-05	2.45E-03	2.9	28.9
antigen processing and presentation of exogenous peptide antigen via MHC class I, TAP-independent (GO:0002480)	4/8	5.72E-05	2.58E-03	31.6	308.3
zymogen activation (GO:0031638)	8/45	6.23E-05	2.73E-03	6.9	66.4
positive regulation of chemotaxis (GO:0050921)	8/45	6.23E-05	2.73E-03	6.9	66.4

(Overlap: # of enriched genes in dataset/ # of genes in term; P-value: Fischer exact test; Adjusted p-value: Benjamini-Hochberg)

Supplementary Table 6:
Individual Study Subjects – Clinical Data and Sample Use

Subject	Treatment group	BMI (kg/m2)	HbA1C (%)			Insulin use (U/kg/d)			MMTT-stimulated C-peptide AUC (pmol/L)			Used in Figure	Used for LC-MS/MS & qPCR
			BL	Y1	Y2	BL	Y1	Y2	BL	Y1	Y2		
1	Verapamil	27.5	6.0	6.3	7.7	0.17	0.19	0.20	0.79	1.31	0.98	1; 2	
2	Verapamil	20.3	6.0	5.4	5.7	0.10	0.13	0.18	0.51	0.66		1; 2; 3	+
3	Verapamil	23.6	6.9	6.6	7.0	0.16	0.22	0.37	0.75	1.06	0.63	1; 2; 3	+
4	Verapamil	28.8	8.7	6.4	6.9	0.42	0.35	0.33	1.23	2.05	2.05	1; 2; 3	+
5	Verapamil	26.0	5.6	6.6	6.6	0.31	0.23	0.23	0.89	0.73	0.81	1; 2	
6	Verapamil/Disc V	20.3	6.5	6.6	7.3	0.19	0.21	0.38	1.11	0.82	0.33	1; 2	
7	Verapamil/Disc V	21.2	7.0	6.4	7.2	0.50	0.70	0.91	0.98	0.99	0.66	1; 2; 3	+
8	Verapamil/Disc V	21.6	6.2	5.9	6.4	0.16	0.22	0.30	0.49	0.39	0.29	1; 2	
9	Verapamil/Disc V	27.4	5.5	5.8	6.2	0.28	0.45	0.37	0.43	0.81	0.68	1; 2; 3	+
10	Control	20.0	6.6	7.1	7.3	0.23	0.32	0.36	0.35	0.13	0.01	1; 2; 3	+
11	Control	22.9	6.1	5.7	6.9	0.18	0.23	0.48	0.66	0.48		1; 2; 3	+
12	Control	22.5	6.9	7.7	8.2	0.18	0.25	0.52	0.65	0.31		1; 2; 3	+
13	Control	27.7	8.7	9.0		0.58	0.69	0.91	0.62	0.35	0.34	1; 2; 3	+
14	Control	26.5	6.4	6.1	6.3	0.27	0.29	0.35	0.43	0.41		1; 2	
15	Control	29.2	7.0	7.7		0.15	0.44		0.75	0.06		1; 2; 3	+

Supplementary Table 7: Primers Used in the Study

Description	Sequences (5'-3')
1. Human GAPDH qPCR 5' primer	ATGGAAATCCCATCACCATCTT
2. Human GAPDH qPCR 3' primer	CGCCCCACTTGATTTTGG
3. Human CD4 qPCR 5' primer	CAAGGTTCTGCCCACATGGT
4. Human CD4 qPCR 3' primer	CAGCACAATCAGGGCCATT
5. Human CXCR3 qPCR 5' primer	TGGCCTGCATCAGCTTTG
6. Human CXCR3 qPCR 3' primer	GGTAGAGCTGGGTGGCATGA
7. Human STAT4 qPCR 5' primer	CCCCTTTGGATTGATGGGTAT
8. Human STAT4 qPCR 3' primer	GCAACAGCCGTTCCTTCTCT
9. Human CXCR5 qPCR 5' primer	CCTGCCACGTTGCACCTT
10. Human CXCR5 qPCR 3' primer	TGAACCAGGCATGCGTTTC
11. Human IL21 qPCR 5' primer	TGGCAACATGGAGAGGATTG
12. Human IL21 qPCR 3' primer	TGGACCAGTGTCCCCAAGA

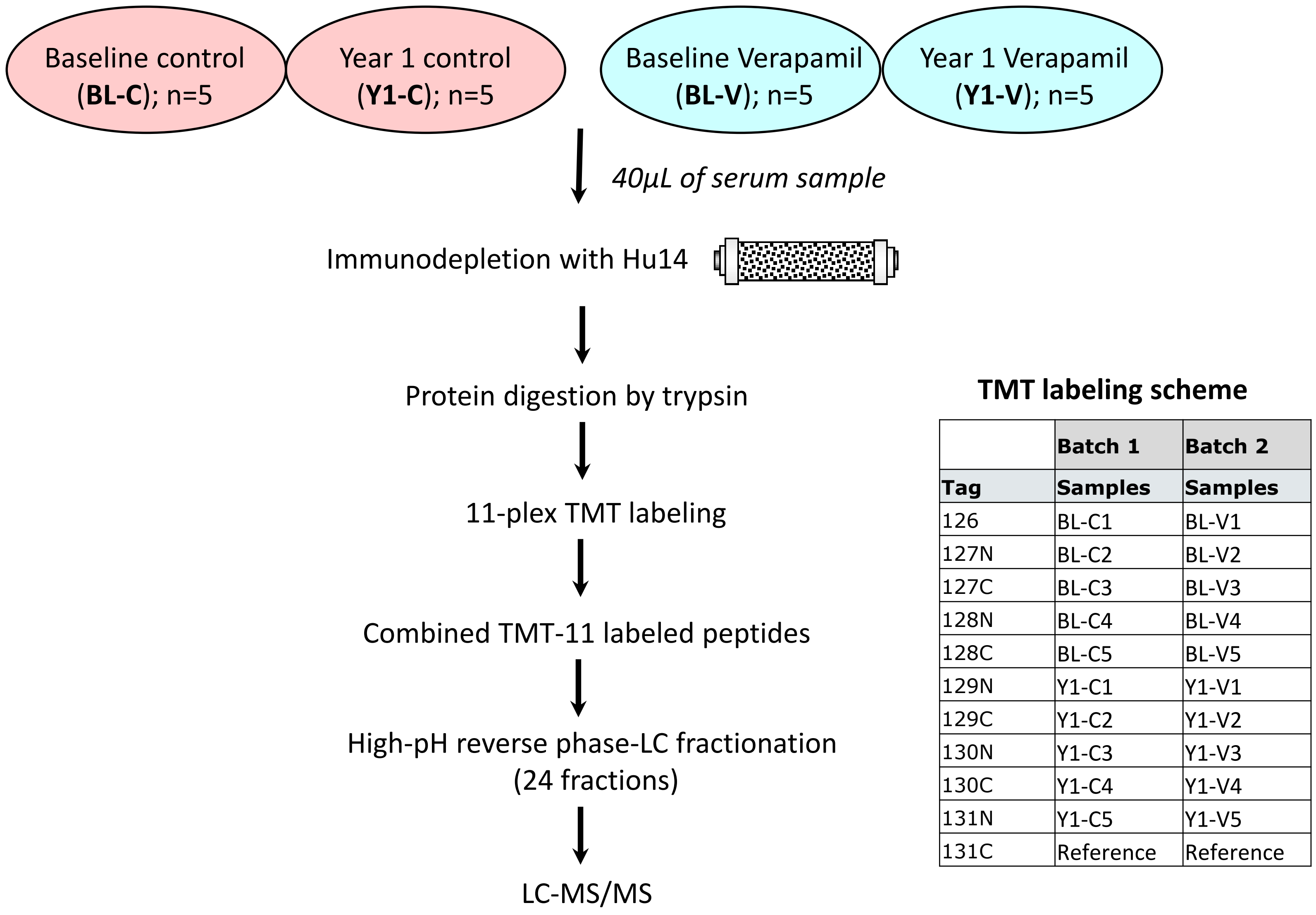

TMT labeling scheme

	**Batch 1**	**Batch 2**
**Tag**	**Samples**	**Samples**
126	BL-C1	BL-V1
127N	BL-C2	BL-V2
127C	BL-C3	BL-V3
128N	BL-C4	BL-V4
128C	BL-C5	BL-V5
129N	Y1-C1	Y1-V1
129C	Y1-C2	Y1-V2
130N	Y1-C3	Y1-V3
130C	Y1-C4	Y1-V4
131N	Y1-C5	Y1-V5
131C	Reference	Reference

Supplementary Figure 1: **Serum proteomics workflow.** Longitudinal serum samples at baseline (BL) and at 1 year follow-up (Y1) from the control (C) and verapamil (V) treatment groups (n=5 pairs from each group) were used for proteomics. Serum samples were depleted by Hu14 column, followed by digestion, TMT labeling, fractionation and LC-MS/MS analyses. The table shows 11-plex TMT labeling scheme for 20 samples in two batch and a pooled reference sample in each batch to facilitate data normalization between TMT plexes.

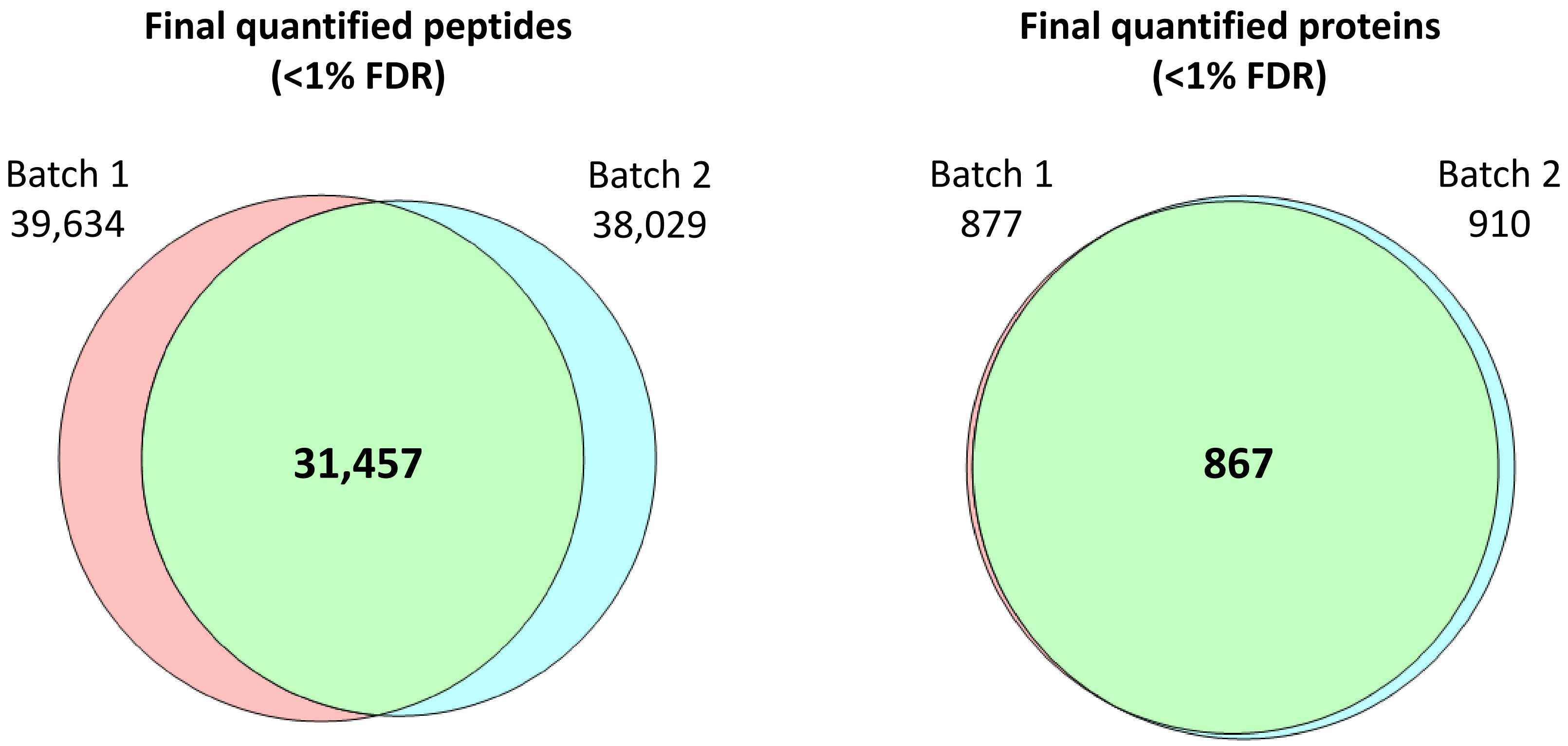

Supplementary Figure 2: **Venn diagrams for final quantified peptides and proteins.** Only peptides and proteins with TMT reporter ion intensity data in all channels from batch 1 (pink) and batch 2 (blue) experiments were considered quantified (green).

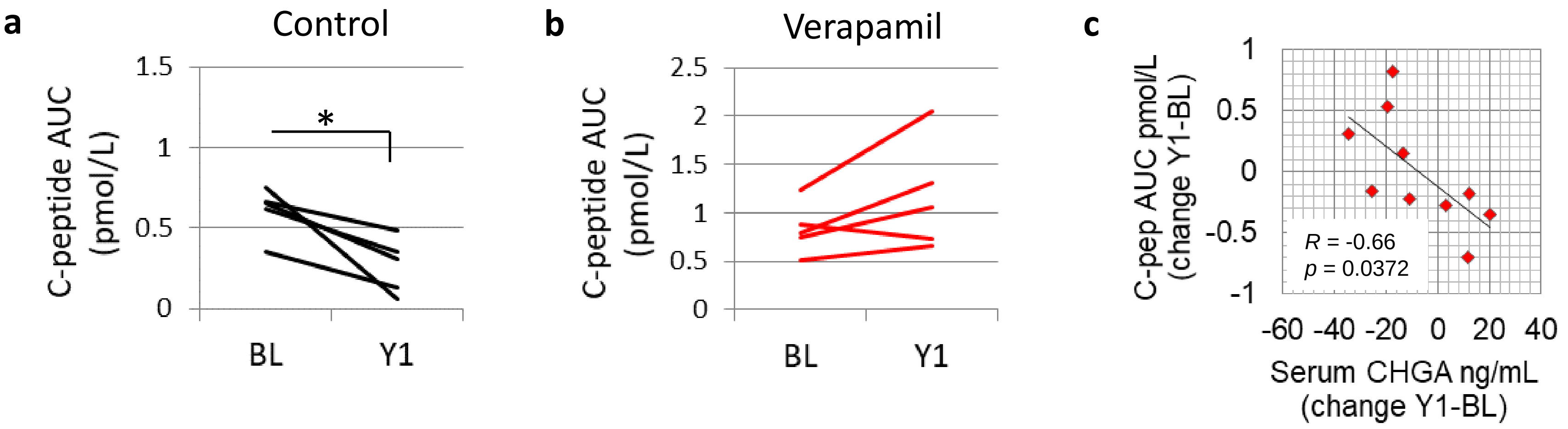

Supplementary Figure 3: **Individual changes in C-peptide AUC and correlation with changes in CHGA.** Stimulated C-peptide AUC at BL and Y1 in individual subjects with T1D receiving placebo (black) (two-tailed, paired Student's t-test: t_4 = 3.673, *P = 0.0213) **(a)** or verapamil (red) **(b)**. Pearson correlation (two-tailed) of the individual changes in C-pep AUC and serum CHGA (R = -0.66, P = 0.0372) **(c)**.

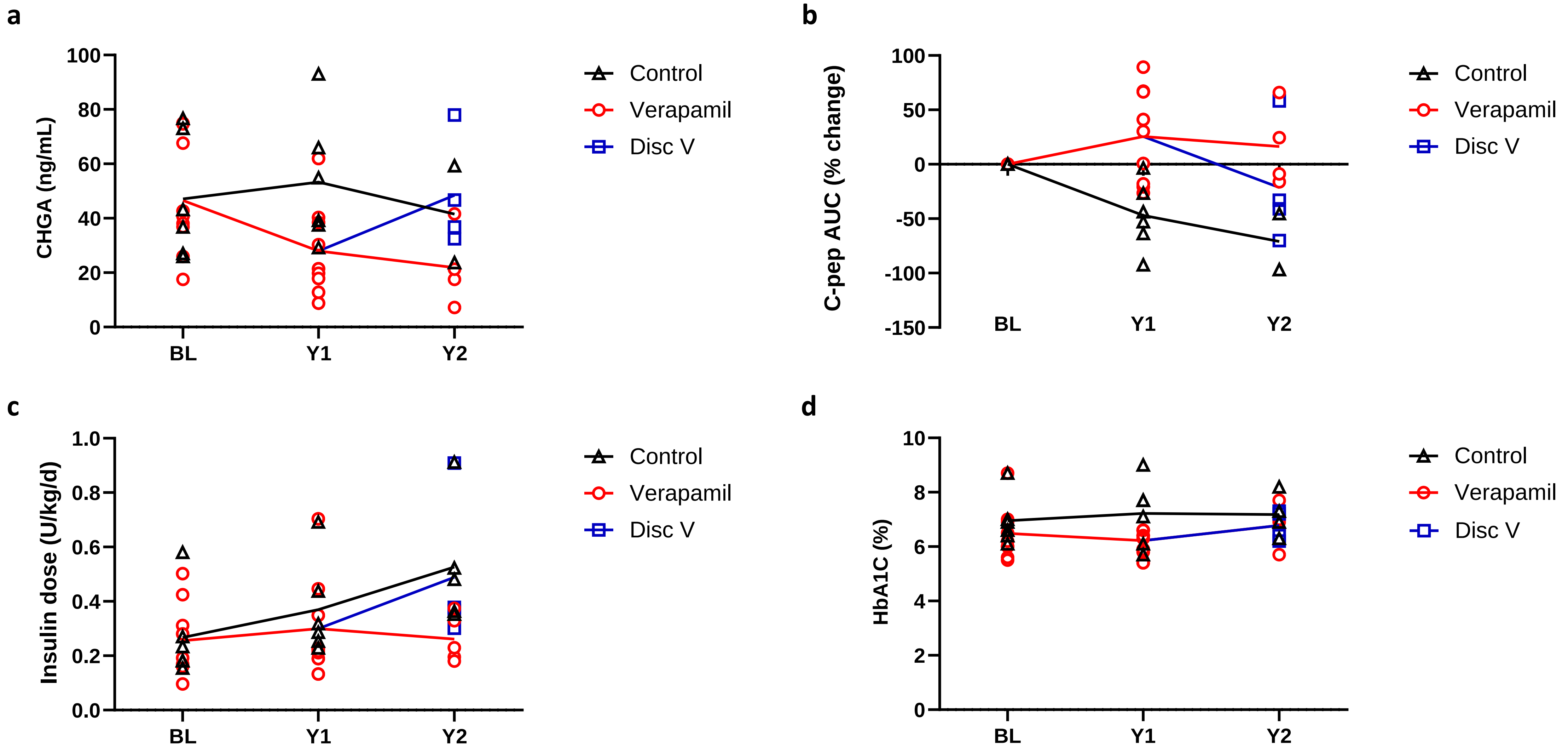

Supplementary Figure 4: **Individual insulin requirements, beta cell function and CHGA over 2 years of T1D treatment with verapamil.** Changes over time in serum CHGA as assessed by ELISA **(a)**, stimulated C-peptide AUC **(b)**, daily insulin dose **(c)** and blood glucose control as assessed by HbA1C **(d)** in subjects with T1D receiving verapamil for 2 years (Verapamil), discontinuing verapamil after the first year (Disc V), or not taking any verapamil (Control). Individual data points are shown and lines represent means.

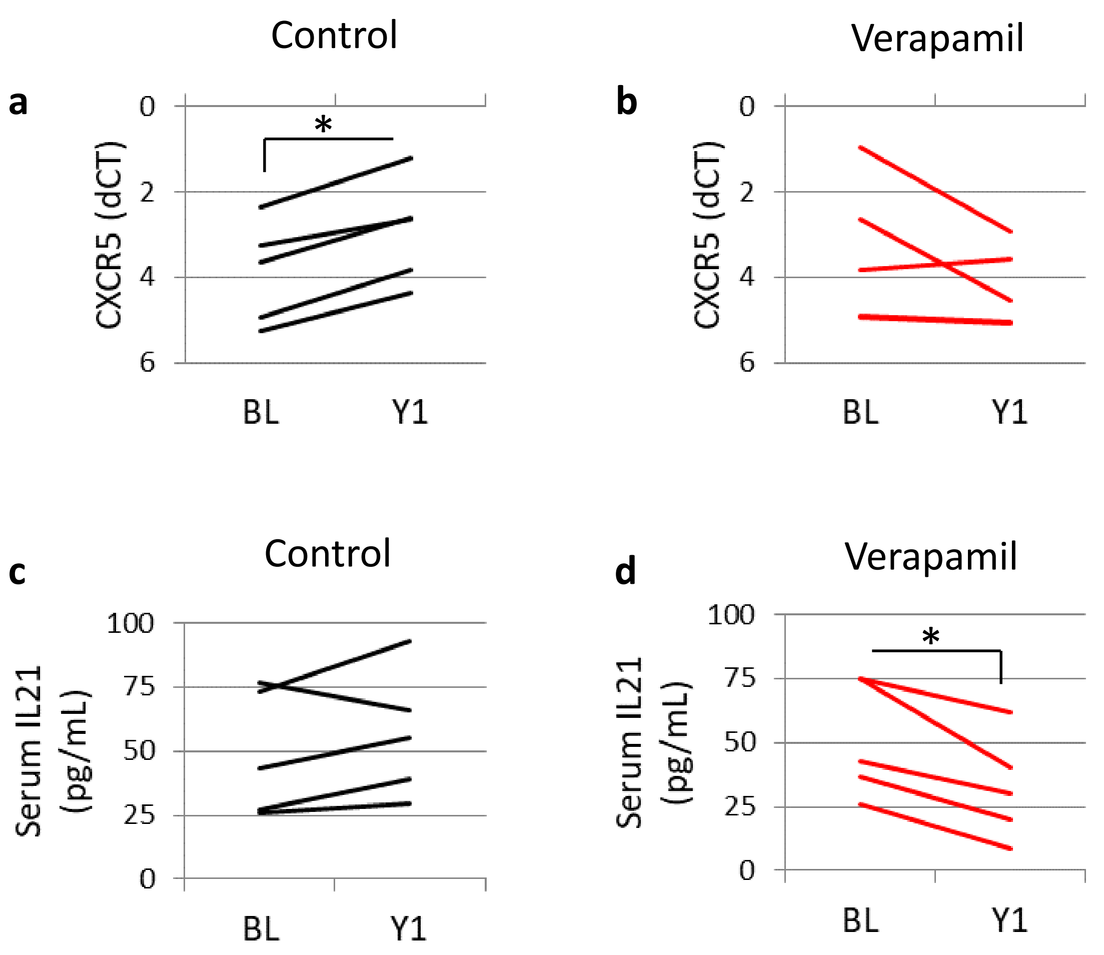

Supplementary Figure 5: Individual changes in CXCR5 and IL-21 in response to verapamil. Expression of *CXCR5* as assessed by qPCR in PBMCs at BL and Y1 in individual subjects with T1D receiving placebo (black) (two-tailed, paired Student's t-test: t_4 = 9.862 , *P = 0.0006) **(a)** or verapamil (red) **(b)**. Individual changes in serum IL21 as assessed by ELISA in subjects receiving placebo **(c)** or verapamil (two-tailed, paired Student's t-test: t_4 = 4.667, *P = 0.0095) **(d).**
