# Supplementary material for: Exploratory study reveals far reaching systemic and cellular effects of verapamil treatment in subjects with type 1 diabetes
Source: Nat Commun. 2022 Mar 3;13:1159. doi: 10.1038/s41467-022-28826-3 (PMC8894430; doi:10.1038/s41467-022-28826-3)
Supplement: Supplementary file 3 — Reporting Summary [file 41467_2022_28826_MOESM3_ESM.pdf]

nature research

Corresponding author(s): Anath Shalev

Last updated by author(s): 2021/11/30

Reporting Summary

Nature Research wishes to improve the reproducibility of the work that we publish. This form provides structure for consistency and transparency in reporting. For further information on Nature Research policies, see our Editorial Policies and the Editorial Policy Checklist.

Please do not complete any field with "not applicable" or n/a. Refer to the help text for what text to use if an item is not relevant to your study.
For final submission: please carefully check your responses for accuracy; you will not be able to make changes later.

Statistics

For all statistical analyses, confirm that the following items are present in the figure legend, table legend, main text, or Methods section.

n/a	Confirmed	
☐	☒	The exact sample size (*n*) for each experimental group/condition, given as a discrete number and unit of measurement
☐	☒	A statement on whether measurements were taken from distinct samples or whether the same sample was measured repeatedly
☐	☒	The statistical test(s) used AND whether they are one- or two-sided<br>*Only common tests should be described solely by name; describe more complex techniques in the Methods section.*
☐	☒	A description of all covariates tested
☐	☒	A description of any assumptions or corrections, such as tests of normality and adjustment for multiple comparisons
☐	☒	A full description of the statistical parameters including central tendency (e.g. means) or other basic estimates (e.g. regression coefficient) AND variation (e.g. standard deviation) or associated estimates of uncertainty (e.g. confidence intervals)
☐	☒	For null hypothesis testing, the test statistic (e.g. F, t, r) with confidence intervals, effect sizes, degrees of freedom and P value noted<br>*Give P values as exact values whenever suitable.*
☒	☐	For Bayesian analysis, information on the choice of priors and Markov chain Monte Carlo settings
☒	☐	For hierarchical and complex designs, identification of the appropriate level for tests and full reporting of outcomes
☐	☒	Estimates of effect sizes (e.g. Cohen's *d*, Pearson's *r*), indicating how they were calculated

Our web collection on statistics for biologists contains articles on many of the points above.

Software and code

Policy information about availability of computer code

Data collection	No software was used for data collection.
Data analysis	SAS 9.4, SigmaStat 4.0, lme4 package [doi:10.18637/jss.v067.i01], R language [https://www.R-project.org/], mzRefinery MS-GF+ v2109.08.26, PlexedPiper [https://github.com/PNNL-Comp-Mass-Spec/PlexedPiper], DESeq2, MASIC (v3.0.7235) [https://github.com/PNNL-Comp-Mass-Spec/MASIC/], Enrichr, STAR (v2.4.2a), Salmon (v0.8.2)

For manuscripts utilizing custom algorithms or software that are central to the research but not yet described in published literature, software must be made available to editors and reviewers. We strongly encourage code deposition in a community repository (e.g. GitHub). See the Nature Research guidelines for submitting code & software for further information.

Data

Policy information about availability of data

All manuscripts must include a data availability statement. This statement should provide the following information, where applicable:

- Accession codes, unique identifiers, or web links for publicly available datasets
- A list of figures that have associated raw data
- A description of any restrictions on data availability

The proteomics data that support the findings of this study have been deposited in ProteomeXchange with accession: PXD026601. The MS raw datasets can also be found in the online repositories: Massive.ucsd.edu with accession: MSV000087598 [https://massive.ucsd.edu/ProteoSAFe/dataset.jsp?task=931c7e16f6ee44be98cbfec8121cc24d].
The transcriptomics data have been deposited in GEO with accession: GSE181328 [https://www.ncbi.nlm.nih.gov/geo/query/acc.cgi?acc=GSE181328]
Publicly available data sets used can be accessed at: [https://www.ncbi.nlm.nih.gov/assembly/GCF_000001405.33/] (H. sapiens reference genome GRCh38.p7) and [https://www.uniprot.org/proteomes/UP000005640] (UniProt human proteome database).

Field-specific reporting

Please select the one below that is the best fit for your research. If you are not sure, read the appropriate sections before making your selection.

☒ Life sciences ☐ Behavioural & social sciences ☐ Ecological, evolutionary & environmental sciences

For a reference copy of the document with all sections, see nature.com/documents/nr-reporting-summary-flat.pdf

Life sciences study design

All studies must disclose on these points even when the disclosure is negative.

Sample size	Sample size was determined by the number of participants in the initial randomized controlled trial (NCT02372253) and the availability of eligible serum samples from the study participants.
Data exclusions	All available data was included in the analysis and no data from adequate samples was excluded.
Replication	All data represent multiple biological replicates as specified in the legends. Based on feasibility and the limited availability of patient samples, experimental replicates were not attempted. Instead, to verify reproducibility and replication, time course experiments and different assays were used.
Randomization	Participants had been randomly allocated into the initial experimental groups.
Blinding	Since this was an exploratory follow up study, the investigators were no longer blinded to the study group allocation of participants, but experimenters and statistician remained blinded to study group allocation of samples.

Reporting for specific materials, systems and methods

We require information from authors about some types of materials, experimental systems and methods used in many studies. Here, indicate whether each material, system or method listed is relevant to your study. If you are not sure if a list item applies to your research, read the appropriate section before selecting a response.

Materials & experimental systems

n/a	Involved in the study	
☒	☐	Antibodies
☒	☐	Eukaryotic cell lines
☒	☐	Palaeontology and archaeology
☒	☐	Animals and other organisms
☐	☒	Human research participants
☐	☒	Clinical data
☒	☐	Dual use research of concern

Methods

n/a	Involved in the study	
☒	☐	ChIP-seq
☒	☐	Flow cytometry
☒	☐	MRI-based neuroimaging

Human research participants

Policy information about studies involving human research participants

Population characteristics	Population characteristics of the the human research participants were well balanced in the experimental groups: age (30 ±2 vs 28 ±3 years), gender (M/F 5/4 vs 3/3), BMI (24.1 ±1.1 vs 24 .8±1.3) and HbA1c (6.5 ±0.3 vs 6.9 ±0.4) (Supplemental Table S1) and all subjects with T1D had been diagnosed within 3 months and were treated with standard insulin therapy.
Recruitment	For this study we used eligible samples from subjects that had been recruited into the initial double-blind randomized controlled trial.
Ethics oversight	University of Alabama at Birmingham (UAB) Internal Review Board

Note that full information on the approval of the study protocol must also be provided in the manuscript.

Clinical data

Policy information about clinical studies

All manuscripts should comply with the ICMJE guidelines for publication of clinical research and a completed CONSORT checklist must be included with all submissions.

Clinical trial registration	NCT02372253

Study protocol	clinicaltrials.gov/ct2/show/NCT02372253
Data collection	Study data was collected at UAB Endocrine Clinics, Birmingham, Alabama from 2/2015- 12/2019.
Outcomes	In the initial trial the primary outcomes measure was defined as the functional beta cell mass determined by area under the curve from a 2-hour mixed meal-stimulated C-peptide and secondary outcomes measures included change in exogenous insulin requirements and HbA1c.

This checklist template is licensed under a Creative Commons Attribution 4.0 International License, which permits use, sharing, adaptation, distribution and reproduction in any medium or format, as long as you give appropriate credit to the original author(s) and the source, provide a link to the Creative Commons license, and indicate if changes were made. The images or other third party material in this article are included in the article's Creative Commons license, unless indicated otherwise in a credit line to the material. If material is not included in the article's Creative Commons license and your intended use is not permitted by statutory regulation or exceeds the permitted use, you will need to obtain permission directly from the copyright holder. To view a copy of this license, visit http://creativecommons.org/licenses/by/4.0/
